# Smoking-Related DNA Methylation is Associated with DNA Methylation Phenotypic Age Acceleration: The Veterans Affairs Normative Aging Study

**DOI:** 10.3390/ijerph16132356

**Published:** 2019-07-03

**Authors:** Yang Yang, Xu Gao, Allan C. Just, Elena Colicino, Cuicui Wang, Brent A. Coull, Lifang Hou, Yinan Zheng, Pantel Vokonas, Joel Schwartz, Andrea A. Baccarelli

**Affiliations:** 1 School of Public Health, Li Ka Shing Faculty of Medicine, The University of Hong Kong, Hong Kong, China; 2 Laboratory of Environmental Epigenetics, Department of Environmental Health Sciences Epidemiology, Columbia University Mailman School of Public Health, New York, NY 10032, USA; 3 Department of Environmental Medicine and Public Health, Icahn School of Medicine at Mount Sinai, New York, NY 10029, USA; 4 Department of Environmental Health, Harvard T.H. Chan School of Public Health, Boston, MA 02115, USA; 5 Department of Biostatistics, Harvard T.H. Chan School of Public Health, Boston, MA 02115, USA; 6 Department of Preventive Medicine, Feinberg School of Medicine, Northwestern University, Chicago, IL 60611, USA; 7 Veterans Affairs Normative Aging Study, Veterans Affairs Boston Healthcare System, Department of Medicine, Boston University School of Medicine, Boston, MA 02118, USA

**Keywords:** smoking-related DNA methylation, DNA methylation phenotypic age, aging biomarker, aging acceleration

## Abstract

DNA methylation may play a critical role in aging and age-related diseases. DNA methylation phenotypic age (DNAmPhenoAge) is a new aging biomarker and predictor of chronic disease risk. While smoking is a strong risk factor for chronic diseases and influences methylation, its influence on DNAmPhenoAge is unknown. We investigated associations of self-reported and epigenetic smoking indicators with DNAmPhenoAge acceleration in a longitudinal aging study in eastern Massachusetts. DNA methylation was measured in whole blood samples from multiple visits for 692 male participants in the Veterans Affairs Normative Aging Study during 1999–2013. Acceleration was defined using residuals from linear regression of the DNAmPhenoAge on the chronological age. Cumulative smoking (pack-years) was significantly associated with DNAmPhenoAge acceleration, whereas self-reported smoking status was not. We observed significant validated associations between smoking-related loci and DNAmPhenoAge acceleration for 52 CpG sites, where 18 were hypomethylated and 34 were hypermethylated, mapped to 16 genes. The *AHRR* gene had the most loci (N = 8) among the 16 genes. We generated a smoking aging index based on these 52 loci, which showed positive significant associations with DNAmPhenoAge acceleration. These epigenetic biomarkers may help to predict age-related risks driven by smoking.

## 1. Introduction

DNA methylation is an aging biomarker that has attracted growing attention in recent years. Tobacco smoking is a leading risk factor for many age-related diseases, including cardiopulmonary diseases and various types of cancers [1]. Several studies have revealed, by means of regulating gene expression and genome stability, that DNA methylation is associated with smoking and smoking-related diseases [2]. Smoking-related CpG sites, located in genes such as *AHRR*, *GPR15*, and *F2RL3*, have been reported in an increasing number of epigenome-wide association studies (EWAS) using whole blood samples [3,4]. A recent study generated a smoking index based on 1501 smoking-related loci, suggesting that such methylation indices could be helpful to predict smoking-related health risks [5]. 

Smoking is a risk factor of disproportional aging that accelerates epigenetic age and decreases life expectancy [6,7]. Aging is characterized by a series of changes at both the cellular and molecular levels, including telomere length shortening, senescence, and variations in gene expression levels. Hannum et al. [8,9] and Horvath (2013) developed the first generation of algorithms to estimate DNA methylation age, which correlates well with chronological age. However, those methods only capture CpG sites that correlate with chronological age and fail to account for an individual’s physiological status [10]. 

A newly developed epigenetic biomarker, DNA methylation phenotypic age (DNAmPhenoAge), was derived from a series of clinical measurements [11,12,13,14,15] to offer a better biomarker of biological aging as a substitute measurement of “phenotypic aging”. DNAmPhenoAge acceleration is defined as a discrepancy between DNAmPhenoAge and chronological age and has been proposed as an indicator of disproportionate aging.

A recent study associated DNAmPhenoAge with self-reported smoking status [10]. However, no study has investigated the association of smoking-related DNA methylation with DNAmPhenoAge or age acceleration. In the current study, we used a well-established cohort to strengthen and extend prior findings with respect to the association between self-reported and epigenetic smoking indicators and age acceleration in whole blood samples.

## 2. Materials and Methods

### 2.1. Study Subjects

This study included 692 participants from the Normative Aging Study (NAS), a closed longitudinal study of aging in men from eastern Massachusetts [16]. A total of 1596 participants free of chronic diseases have been enrolled in NAS since 1984, and they undergo physical examinations every 3–5 years. At each visit, information on demographic factors, lifestyle, and disease history were collected, and physical examinations and laboratory tests were performed. Specifically, we analyzed DNA samples up to 4 visits from 1999–2013. The current study was restricted to the first three visits to observe long-term DNAmPhenoAge acceleration and control for race heterogeneity. Participants provided written informed consent during each visit, and the Veterans Affairs Boston Health Care System Institutional Review Board approved the study (code: AAAQ8739).

### 2.2. DNA Methylation Data

DNA was extracted from stored blood buffy coats using the QIAamp DNA Blood Kit (Qiagen, Valencia, CA, USA). We treated 500 ng DNA for bisulfite conversion using the EZ-96 DNA Methylation Kit (Zymo Research, Orange, CA, USA). After bisulfite conversion, we hybridized DNA samples to 12-sample Illumina HumanMethylation450 BeadChips per Infinium HD Methylation protocol (Illumina, San Diego, CA, USA). Technical effects resulting from plates/chips were minimized with a two-stage, age-stratified algorithm to randomize the samples. The β-values, ranging from 0–1, were used to represent the methylation status of a specific CpG site, with 0 indicating no methylation and 1 indicating full methylation. 

Calculation of DNAmPhenoAge: DNAmPhenoAge was estimated using the method described by Levine et al. [10]. In short, phenotypic age was developed using clinical measurements from the third National Health and Nutrition Examination Survey. A penalized Cox regression model was used to select 10 out of 42 biomarkers. Phenotypic age was then regressed on DNA methylation data from whole blood samples based on an elastic net regression analysis, which led to 513 CpG sites. We calculated DNAmPhenoAge based on these loci for each participant as:

DNAmPhenoAge = CpG_1_ × *β*_1_ + CpG_2_ × *β*_2_ + CpG_3_ × *β*_3_ +…+ CpG_513_ × *β*_513_ + intercept (1)

Values of coefficients and intercepts were extracted from Levine et al. [10]. DNAmPhenoAge acceleration was defined as residuals from a linear regression of DNAmPhenoAge on chronological age.

Selection of smoking-related DNA methylation loci: We selected 151 CpG sites reported from previous smoking EWAS at least twice from a previous systematic review [3]. There was no overlap between these 151 loci and the 513 loci used to predict DNAmPhenoAge. 

### 2.3. Statistical Analysis

**Figure 1:**
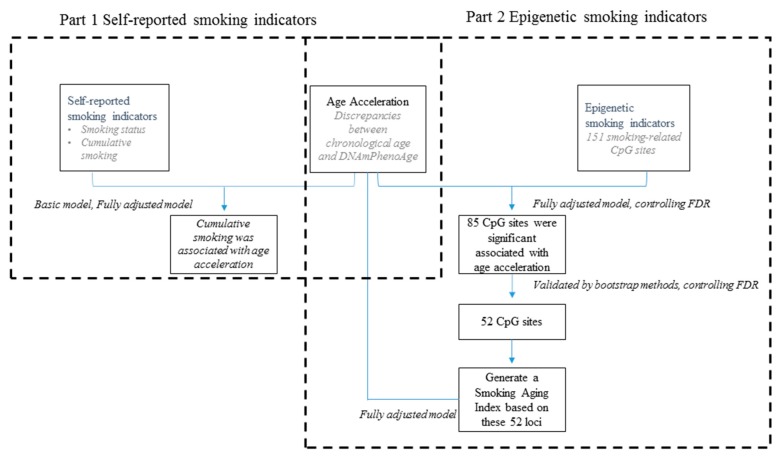
Flow chart of the generalized linear mixed model used to assess the relationship between smoking indicators and DNA methylation phenotypic age (DNAmPhenoAge) acceleration of participants in the Veterans Affairs Normative Aging Study in Boston, MA, 1999–2013.

Descriptive statistics were used to present distributions of demographic and clinical variables among all participants. Distributions of these variables across each visit were described as well. Generalized linear mixed models were used to assess the relationship between smoking indicators and DNAmPhenoAge acceleration (Figure 1). We conducted two parts of statistical analysis: first, to explore the association between self-reported smoking indicators and DNAmPhenoAge acceleration; and second, to explore the association between epigenetic smoking indicators and DNAmPhenoAge acceleration.

First, we examined the associations between DNAmPhenoAge acceleration and self-reported smoking indicators smoking status (never, current, or former) and cumulative smoking status (pack-years). Model 1 was adjusted for age (years) and leukocyte distribution was estimated using the Houseman et al.’s algorithm [17]. In addition, both visits and random batch effects of methylation measurement were included in the model as random effects. Additionally, Model 2 was adjusted for smoking status, alcohol consumption (abstainer, low, intermediate, or high), body mass index (BMI) (underweight or normal weight BMI <25, overweight BMI ≥25 to <30, or obese BMI ≥30), physical activity (low, metabolic equivalent of task (MET) ≤12 kcal/kg hours/week; median, MET 12–30 kcal/kg hours/week; or high, MET ≥30 kcal/kg hours/week), years of education (≤12 years, 13–16 years, or >16 years), hypertension, stroke, coronary heart disease, and diabetes (yes/no).

Second, we tested if epigenetic indicators were associated with DNAmPhenoAge acceleration. The aforementioned fully adjusted model (Model 2) was used to explore associations between DNAmPhenoAge acceleration and each of the 151 smoking-related loci. False discovery rate (FDR)-adjusted [18] *P*-values were used to account for multiple testing. The alpha threshold for FDR-adjusted *P*-value was set at ≤0.05. We performed bootstrap analysis to validate the selected CpGs. We resampled the original dataset to create 1000 simulated datasets for each locus. Then, we ran the model in each dataset and returned the effect estimates for the tested CpG sites. We counted the effect estimates that were greater than or equal to the observed value and divided that count by the number of estimates. We again used FDR-adjusted *P*-values to select the most robust loci. 

Finally, we generated a smoking aging index based on the verified 52 CpG sites [5]. The mean β-value (*μ_c_*) and standard deviation (SD) (*σ_c_*) were calculated across the never-smokers for each selected CpG. The smoking aging index was defined as

(2) $$\mathrm{SI}\left(s\right)=\;\frac{1}{n}\overset{n}{\underset{c}{\sum }}W_{c}\frac{\beta_{cs}-\mu_{c}}{\sigma_{c}}$$

where, W_c_ is +1 if the smoking-associated CpG, *c*, is hypermethylated or −1 if it is hypomethylated in smokers, and β_c_ is the β-value of this CpG in sample *s* [5]. The smoking aging index for each participant was calculated based on these validated DNAmPhenoAge acceleration-related loci, which were validated by the bootstrap method. Statistical analysis was performed using R 3.5.1 (R core team, 2018).

## 3. Results

### 3.1. Characteristics of Participants

**Figure 2:**
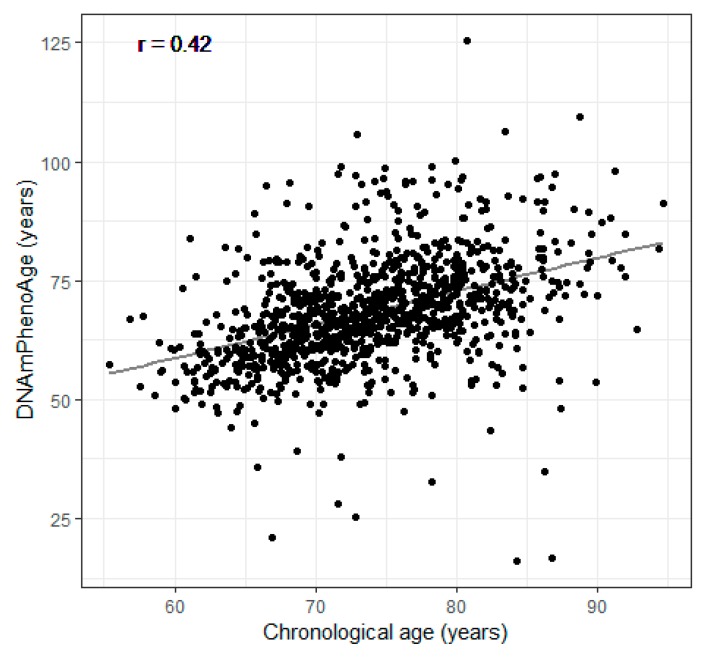
Scatter plot of the DNA methylation phenotypic age (DNAmPhenoAge) and the chronological age of participants in the Veterans Affairs Normative Aging Study in Boston, MA, 1999–2013.

Demographic and clinical characteristics of the participants at all three visits are shown in Table 1. Participants were 55–94 years old with a mean (SD) age of 72.7 (6.7) years at first visit. The mean (SD) of the DNAmPhenoAge was 68.4 (11.9) years at first visit, younger than the chronological age. The chronological age and the DNAmPhenoAge were correlated with each other (r = 0.42, *P* < 0.001) (Figure 2). The majority of the participants in the cohort were former smokers and never smokers, accounting for 66% and 30% at the first visit, respectively. The percentage for current smokers was only 4% at the first visit. The total years of smoking was 16.9 years on average at the first visit. 

### 3.2. DNAmPhenoAge Acceleration and Smoking Indicators

**Figure 3:**
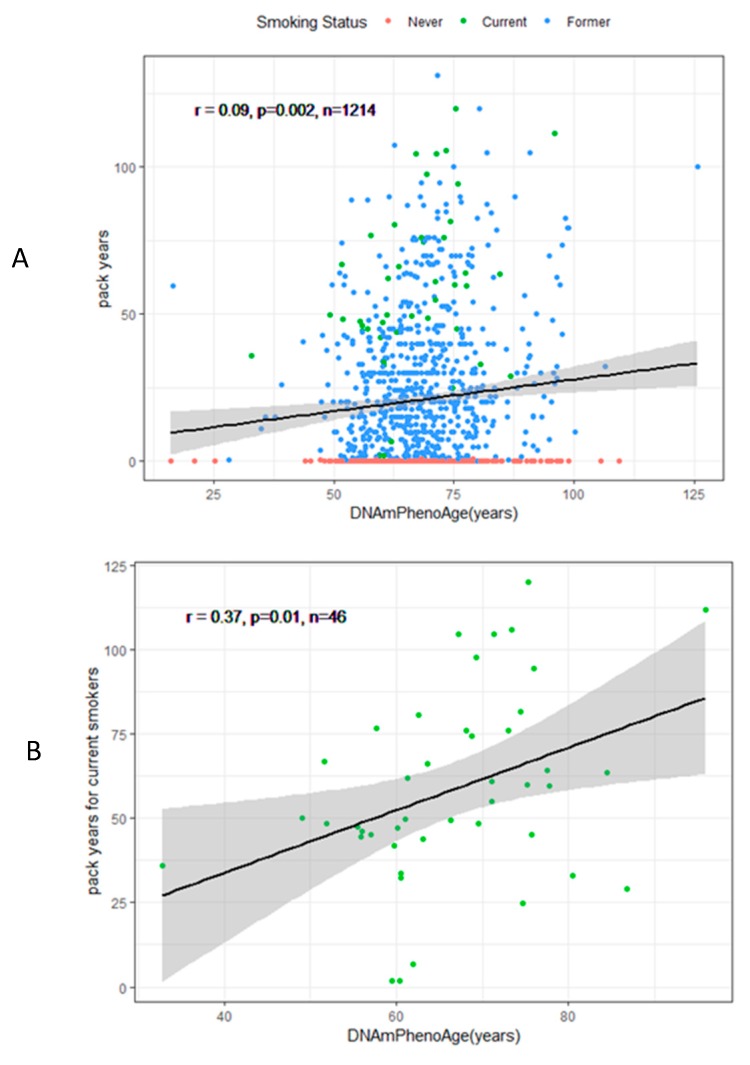

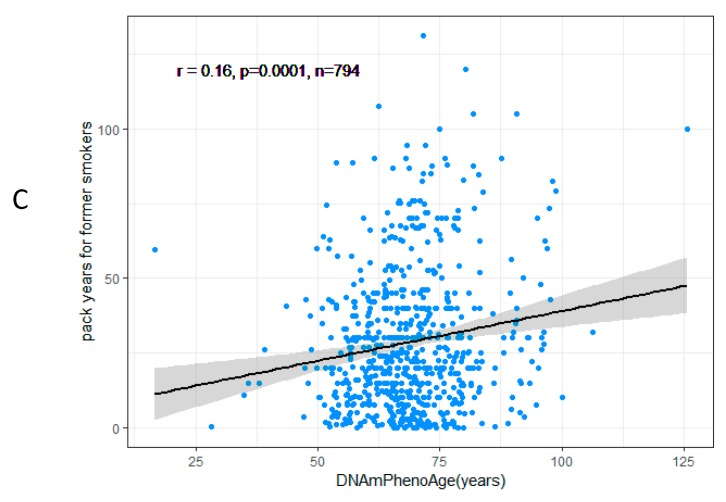
Scatter plots of the association between pack-years and DNA methylation phenotypic age (DNAmPhenoAge) for (**A**) all participants, (**B**) current smokers, and (**C**) former smokers in the Veterans Affairs Normative Aging Study in Boston, MA, 1999–2013.

Table 2 illustrates the association of DNAmPhenoAge acceleration with self-reported (smoking status and pack-years) indicators in both the basic model (Model 1) and fully adjusted model (Model 2). Only current smokers had significantly higher DNAmPhenoAge acceleration in Model 1, although not in Model 2. However, cumulative smoking (pack-years) was significantly associated with DNAmPhenoAge acceleration in both models. Figure 3 shows associations between DNAmPhenoAge and pack-years for all participants (r = 0.09, N = 1214, *P* = 0.002), current smokers (r = 0.37, N = 46, *P* = 0.01), and former smokers (r = 0.16, N = 794, *P* = 0.0001).

Moreover, we assessed associations between the 151 smoking-related CpG candidates and DNAmPhenoAge acceleration using Model 2, with visits and random batch effects of methylation measurement as random effects. A total of 85 of 151 CpG candidates passed the threshold of FDR <0.05, and thus demonstrated significant associations with DNAmPhenoAge acceleration. We attempted to validate theses CpG candidates using bootstrap methods. Validation confirmed that 52 of 85 CpG sites were associated with DNAmPhenoAge acceleration (FDR < 0.05, Table 3). Of these 52 loci, 18 were hypomethylated and 34 were hypermethylated. *AHRR* had the most related loci (N = 8). 

We generated a smoking aging index based on the 52 validated smoking-related loci and compared this new index with *AHRR* cg05575921, a strong smoking-associated hypomethylated locus. The smoking aging index was highly associated with self-reported smoking indicators in the generalized linear mixed effect model (*P* < 0.05, data not shown). For cg05575921, negative associations were found in both Model 1 (β = −1.34, *P* = 0.0001) and Model 2 (β = −0.97, *P* = 0.007) (Table 3). For the smoking aging index, positive associations were found in both Model 1 (*β* = 1.92, *P* < 0.0002) and Model 2 (*β* = 1.91, *P* < 0.0001). 

## 4. Discussion

We systematically explored the association between self-reported and epigenetic smoking indicators and DNAmPhenoAge in whole blood samples based on an elderly cohort in eastern Massachusetts. One of the self-reported variables of smoking, cumulative smoking expressed in pack-years, was significantly and strongly associated with DNAmPhenoAge acceleration. For epigenetic indicators, 52 previously confirmed CpG sites (including a robust mono-biomarker of smoking, ch05575921) and smoking aging index derived from these loci were associated with DNAmPhenoAge acceleration. 

Tobacco smoking has long been regarded as a leading risk factor for several age-related health outcomes [1,19,20]. A recent study investigated the relationship between DNAmPhenoAge acceleration based on Horvath’s and Hannum et al.’s algorithms and smoking-related DNA methylation [21]. Although our current study assessed DNAmPhenoAge acceleration based on DNAmPhenoAge, our findings are consistent with those of Gao et al., since we both found loci mapping to *AHRR*, a well-known tumor suppressor gene. One specific *AHRR* locus, cg05575921, has been regarded as a strong epigenetic smoking indicator in previous smoking EWAS [22,23,24] and was confirmed by our current study [25]. Other consistent loci involve *CNTNAP2* (contactin associated protein-like 2) and have been reported associated with several mental diseases [26,27,28], as well as *KCNQ1* (voltage-gated KQT-like subfamily Q, member 1) that has been associated with type 2 diabetes [29]. Moreover, our current study also unveiled other genes, such as *GNG12* (a negative regulator of inflammation in microglial cells that could impact the neural system) [30], *LRRN3* (a member of neuron leucine-rich repeat superfamily that may be associated with neural system development and differentiation processes) [31], and *GFI1* (involved in the regulation of hematopoietic stem cells) [32].

There are some inconsistencies between self-reported smoking indicators and epigenetic smoking indicators with respect to their associations with aging. One inconsistency is that the smoking status was not related to either DNAmPhenoAge acceleration in the fully-adjusted model (Table 2) or the smoking aging index (Figure 3). One explanation is that the number of current smokers is far less than former and never smokers, which may diminish statistical power. Another reason might be the susceptibility of different individuals. For instance, genetic polymorphisms may play a role in modifying the health impacts of exposure to environmental health hazards, indicating that some individuals may be more or less likely to be affected by environmental hazards and develop diseases [33,34]. 

This point leads to our consideration that genetic variation may affect the relationship between epigenetics and aging [35]. A recent study reported findings regarding the heritability of discrepancies between chronological and epigenetic age by examining twins [9]. Moreover, we should also bear in mind that exposure to cigarette smoking is not the only factor that could lead to differences in DNA methylation. Other environmental hazards, such as drinking alcohol, lifestyle or nutritional factors, or interaction effects between these hazards and smoking, might induce similar methylation changes as well [36,37]. This evidence supports the idea that aging rates are influenced by interactions among genetic variation, environmental exposures, and the epigenome. 

Our study has the following strengths. We present a longitudinal study to investigate the relationship between smoking-related DNA methylation and aging. Longitudinal studies that investigate aging have advantages over cross-sectional studies, particularly with regard to genetic variations [38]. Furthermore, the cohort longitudinal design allowed us to understand relationships between genetics and other factors that specify inter-subject temporal changes in human DNA methylation profiles.

However, there are also limitations to our study. First, we validated our results using a bootstrap method in the training cohort instead of an external validation cohort. Although it is generally ideal to use another cohort to validate and replicate findings, we could not access another similar phenotypic cohort. Therefore, further studies are needed to validate and replicate our findings. Second, our signature to predict smoking-related aging was derived from elderly males only. Thus, it remains unclear whether the signature would differ in a female population. However, previous EWAS suggest that smoking-associated changes in blood samples are not dependent on sex [39,40]. Nonetheless, future validation in a larger sex-balanced cohort is still needed. Third, measurement inaccuracies may affect the comparability of longitudinal DNAmPhenoAge acceleration. However, we adjusted for batch effect by including it as a random effect in the model and normalized methylation profiles using Horvath’s internal normalization methods. Fourth, loss to follow-up may have biased our findings. However, we used inverse probability weighting to impute missing data as a sensitivity analysis.

## 5. Conclusions

In summary, our results show that self-reported cumulative smoking and epigenetic smoking indicators were associated with DNAmPhenoAge acceleration. Epigenetic changes at specific smoking-related CpG sites and our combined smoking aging index might be useful biomarkers to predict smoking-associated aging. Further research is warranted to replicate our findings in follow-up studies and to identify the mechanism underlying the effects of smoking on DNAmPhenoAge.

## Figures and Tables

**Table 1:** Population characteristics of participants in the Veterans Affairs Normative Aging Study in Boston, MA, 1999–2013 ^a^.

Variable	All Visits	First Visit	Second Visit	Third Visit
No. of measures	1214	692	418	104
Chronological age (years)	74.3 (7.0)	72.7 (6.7)	76.3 (6.8)	79.8 (6.0)
DNAmPhenoAge (years)	70.5 (12.9)	68.4 (11.9)	70.6 (12.2)	83.6 (13.9)
Smoking status				
Never smoker	374 (31%)	207 (30%)	131 (31%)	36 (35%)
Current smoker	46 (4%)	29 (4%)	15 (4%)	2 (2%)
Former smoker	794 (65%)	456 (66%)	272 (65%)	66 (63%)
Total years of smoking cigarettes (years)	16.7 (15.8)	16.9 (15.7)	16.4 (15.9)	11.3 (15.5)
Alcohol consumption (g/d)				
Abstainer	230 (24%)	146 (24%)	81 (25%)	3 (25%)
Low (0–40 g/d)	652 (69%)	420 (69%)	223 (70%)	9 (75%)
Intermediate (40–60 g/d)	37 (4%)	26 (4%)	11 (3%)	0
High (≥60 g/d)	23(2%)	18 (3%)	5 (2%)	0
Physical activity (MET-hours/week)				
Low(≤12 kcal/kg hours/week)	667 (64%)	408 (63%)	240 (65%)	19 (58%)
Median (12–30 kcal/kg hours/week)	227 (22%)	139 (22%)	83 (23%)	5 (15%)
High (≥30 kcal/kg hours/week)	152 (15%)	98 (15%)	45 (12%)	9 (27%)
Years of education				
≤12 years	335 (33%)	215 (33%)	115 (33%)	5 (42%)
13–16 years	477 (47%)	314 (48%)	159 (46%)	4 (33%)
>16 years	209 (20%)	131 (20%)	75 (21%)	3 (25%)
Body mass index				
Underweight or normal weight (<25.0)	265 (22%)	139 (20%)	94 (22%)	32 (31%)
Overweight (≥25.0 to <30.0)	636 (52%)	374 (54%)	214 (51%)	48 (46%)
Obese (≥30.0)	313 (26%)	179 (26%)	110 (26%)	24 (23%)
Chronic diseases				
Hypertension	731 (71%)	464 (70%)	258 (73%)	9 (75%)
Stroke	99 (8%)	53 (8%)	39 (9%)	7 (7%)
Coronary heart disease	397 (33%)	208 (30%)	146 (35%)	43 (41%)
Diabetes	193(16%)	99 (14%)	73 (17%)	21 (20%)

^a^ Mean value (standard deviation) for continuous variables and *n* (%) for categorical variables.

**Table 2:** Association of DNA methylation phenotypic age (DNAmPhenoAge) acceleration with self-reported smoking and epigenetic smoking indicators in the Veterans Affairs Normative Aging Study Cohort in Boston, MA, 1999–2013.

Smoking Indicator	Model 1 ^a^			Model 2 ^b^		
Smoking Status	Estimate ^#^	SE *	*P*-Value	Estimate ^#^	SE	*P*-Value
Current smoker	3.41	1.83	0.06	2.69	1.86	0.15
Former smoker	0.62	0.77	0.41	0.17	0.79	0.82
Never smoker		Ref			Ref	
Cumulative smoking (pack-year)	0.06	0.01	3.4 e-5	0.04	0.01	0.003

^#^ Effect size of age acceleration per SD (standard deviation) increase of smoking aging index. * SE: standard error. ^a^ Model 1: Adjusted for age (years), leukocyte distribution (Houseman et al.’s algorithm), visits, and random batch effect of methylation measurement. ^b^ Model 2: Additionally adjusted for smoking status, alcohol consumption (abstainer/low/intermediate/high), body mass index (BMI; underweight or normal weight/overweight/obese), physical activity (metabolic equivalent of task (MET) low ≤12 kcal/kg hours/week, MET median 12–30 kcal/kg hours/week, and MET high ≥30 kcal/kg hours/week), years of education (≤12 years, 13–16 years, >16 years), hypertension, stroke, coronary heart disease, and diabetes (yes/no).

**Table 3:** CpG Sites (*P* < 0.05) associated with DNA methylation phenotypic age (DNAmPhenoAge) acceleration in the Veterans Affairs Normative Aging Study Cohort in Boston, MA, 1999–2013 ^a^.

Chr	CpG Site	Gene	Effect Size per SD (SE) ^#^	*P*-Value	FDR- Adjusted *P*-Value	*P*-Value for Bootstrap	FDR-Adjusted *P*-Value
1	cg25189904	*GNG12*	1.21	6.7E−04	2.0E−03	1.1E−02	2.7E−02
	cg04885881		1.91	3.5E−08	4.0E−07	2.7E−08	3.0E−07
	cg11314684	*AKT3*	1.82	4.3E−08	4.6E−07	2.0E−03	7.7E−03
	cg09662411	*GFI1*	−1.66	2.5E−06	1.9E−05	5.0E−03	1.6E−02
	cg10399789	*GFI1*	1.58	4.5E−06	3.0E−05	1.7E−02	3.3E−02
	cg12547807		2.35	1.8E−14	6.9E−13	1.1E−13	4.3E−12
	cg19713429	*CAPZB*	1.89	6.6E−09	8.3E−08	1.0E−03	4.5E−03
	cg21140898		−1.61	7.1E−06	4.1E−05	4.3E−06	1.3E−05
	cg26764244	*GNG12*	2.01	3.1E−09	4.4E−08	2.0E−03	7.7E−03
	cg27537125		2.82	1.1E−19	1.6E−17	5.3E−18	1.3E−16
2	cg01940273		−1.40	2.7E−04	9.2E−04	1.3E−02	2.9E−02
	cg03329539		1.71	2.7E−07	2.5E−06	1.6E−02	3.2E−02
	cg23079012		−1.05	9.8E−04	2.6E−03	1.5E−02	3.0E−02
3	cg18642234	*GPX1*	1.86	2.9E−06	2.1E−05	1.4E−05	1.7E−04
	cg18754985	*CLDND1*	−1.13	1.6E−04	5.9E−04	3.0E−03	1.1E−02
4	cg24556382	*GALNT7*	1.40	3.7E−05	1.7E−04	7.0E−03	2.0E−02
5	cg14817490	*AHRR*	1.85	2.6E−07	2.5E−06	1.5E−06	3.8E−05
	cg05575921	*AHRR*	−0.97	1.3E−05	1.2E−04	7.0E−03	2.0E−02
	cg25648203	*AHRR*	−1.14	6.6E−04	2.0E−03	2.2E−02	4.0E−02
	cg26703534	*AHRR*	−1.54	1.6E−05	7.7E−05	1.5E−02	3.0E−02
	cg01899089	*AHRR*	1.53	6.9E−06	4.1E−05	4.9E−05	1.2E−05
	cg01097768	*AHRR*	2.50	3.4E−12	6.3E−11	2.1E−11	4.5E−10
	cg11554391	*AHRR*	1.72	3.2E−06	2.2E−05	2.0E−03	7.7E−03
	cg17924476	*AHRR*	1.55	4.9E−06	3.1E−05	3.0E−03	1.1E−02
6	cg24859433		−1.44	5.8E−05	2.4E−04	1.5E−02	3.0E−02
	cg14753356		1.65	5.1E−04	1.6E−03	1.2E−02	2.8E−02
	cg15474579	*CDKN1A*	1.68	1.0E−05	5.5E−05	2.7E−02	4.7E−02
	cg20778199		−3.01	5.7E−17	4.3E−15	3.6E−16	2.7E−14
7	cg11207515	*CNTNAP2*	1.72	5.0E−07	4.3E−06	5.0E−03	1.6E−02
	cg25949550	*CNTNAP2*	2.56	8.7E−14	2.2E−12	6.0E−13	1.4E−11
	cg05221370	*LRRN3*	2.43	6.6E−12	1.1E−10	4.9E−11	4.3E−09
	cg07826859	*MYO1G*	1.64	4.6E−05	2.0E−04	2.8E−02	4.8E−02
	cg09837977	*LRRN3*	1.92	1.6E−05	7.7E−05	9.0E−03	2.4E−02
8	cg25305703		1.39	6.7E−05	2.7E−04	1.3E−02	2.9E−02
10	cg25953130	*ARID5B*	−1.54	1.5E−05	7.6E−05	7.0E−03	2.0E−02
11	cg23771366	*PRSS23*	1.31	8.1E−04	2.4E−03	1.5E−02	3.0E−02
	cg04039799	*NAV2*	1.85	3.2E−09	4.4E−08	1.4E−08	3.9E−07
	cg16556677	*KCNQ1*	−1.41	2.2E−04	7.7E−04	8.0E−03	2.2E−02
	cg16611234		1.36	1.7E−04	5.9E−04	7.0E−03	2.0E−02
12	cg02583484	*HNRNPA1*	1.22	3.5E−04	1.2E−03	2.0E−02	3.8E−02
	cg04158018	*NFE2*	−1.38	1.1E−04	4.0E−04	4.0E−03	1.4E−02
13	cg23681440		1.17	8.9E−04	2.5E−03	2.4E−02	4.3E−02
14	cg13976502	*C14orf43*	1.22	1.5E−05	7.6E−05	1.0E−03	4.5E−03
	cg22851561	*C14orf43*	2.49	1.0E−15	5.2E−14	3.7E−14	6.4E−13
	cg13038618		1.20	1.1E−03	2.9E−03	2.9E−02	4.8E−02
16	cg06972908	*ITGAL*	2.64	2.5E−14	7.7E−13	9.3E−12	4.6E−10
	cg13500388	*CBFB*	1.67	1.7E−05	7.7E−05	1.1E−02	2.7E−02
	cg16794579	*XYLT1*	2.37	6.7E−13	1.4E−11	3.5E−12	6.9E−10
17	cg19572487	*RARA*	1.43	7.2E−05	2.8E−04	2.2E−02	4.0E−02
	cg07465627	*STXBP4*	1.46	1.8E−06	1.4E−05	6.2E−05	7.1E−04
19	cg15187398	*MOBKL2A*	1.84	5.1E−07	4.3E−06	4.2E−06	1.8E−05
	cg23973524	*CRTC1*	−1.63	7.4E−06	4.1E−05	1.0E−02	2.6E−02

^#^ SD: Standard deviation; SE: Standard error. ^a^ Adjusted for age (years), leukocyte distribution (Houseman et al.’s algorithm), smoking status, alcohol consumption (abstainer/low/intermediate/high), body mass index (BMI; underweight or normal weight/overweight/obese), physical activity (metabolic equivalent of task (MET) low ≤12 kcal/kg hours/week, MET median 12–30 kcal/kg hours/week, and MET high ≥30 kcal/kg hours/week), years of education (≤12 years, 13–16 years, >16 years), hypertension, stroke, coronary heart disease, diabetes, cancer (yes/no), visits, and random batch effect of methylation measurement.
